# Significance of the oceanic CO_2 _sink for national carbon accounts

**DOI:** 10.1186/1750-0680-1-5

**Published:** 2006-07-21

**Authors:** Ben I McNeil

**Affiliations:** 1 Climate & Environmental Dynamics Laboratory, School of Mathematics, University of New South Wales, Sydney, NSW, Australia

## Abstract

**Background:**

Under the United Nations convention on the law of the sea (1982), each participating country maintains exclusive economic and environmental rights within the oceanic region extending 200 nm from its coastline, known as the Exclusive Economic Zone (EEZ). Although the ocean within each EEZ has a vast capacity to absorb anthropogenic CO_2 _and therefore potentially be used as a carbon sink, it is not mentioned within the Kyoto Protocol most likely due to inadequate quantitative estimates. Here, I use two methods to estimate the anthropogenic CO_2 _storage and uptake for a typically large EEZ (Australia).

**Results:**

Depending on whether the Antarctic territory is included I find that during the 1990s between 30–40% of Australia's fossil-fuel CO_2 _emissions were absorbed by its own EEZ.

**Conclusion:**

This example highlights the potential significance of the EEZ carbon sink for national carbon accounts. However, this 'natural anthropogenic CO_2 _sink' could be used as a disincentive for certain nations to reduce their anthropogenic CO_2 _emissions, which would ultimately dampen global efforts to reduce atmospheric CO_2 _concentrations. Since the oceanic anthropogenic CO_2 _sink has limited ability to be controlled by human activities, current and future international climate change policies should have an explicit 'EEZ' clause excluding its use within national carbon accounts.

## Background

Atmospheric CO_2 _concentrations would be about 55 ppm (parts per million) higher than their present concentration without the oceanic anthropogenic CO_2 _sink. The ocean has hindered the extent of accelerated climate change and will continue to absorb about 33% of fossil-fuel emissions well into the future [1]. The ocean CO_2 _sink is different to other carbon sinks in that it directly remediates against climate change by sequestering *anthropogenic *CO_2 _on both short and long timescales. The exclusive economic zone (EEZ) is an oceanic zone legally bound to nation states under international law [2]. With the global EEZ representing 27% of the oceans area, the question arises as to how significant could the EEZ CO_2 _sink be for national carbon accounts. I use Australia as a case study to estimate the EEZ anthropogenic CO_2 _sink due largely to the detailed accounting information on CO_2 _emissions from fossil fuels and land-use changes [3] along with its very large oceanic territory. In fact excluding the Antarctic territorial claim, Australia's EEZ is one of the largest in the world and covers an area 8.2 × 10^6 ^km^2 ^[4], which makes it larger than its continental land area (7.7 × 10^6 ^km^2^).

## Results and discussion

**Figure 1:**
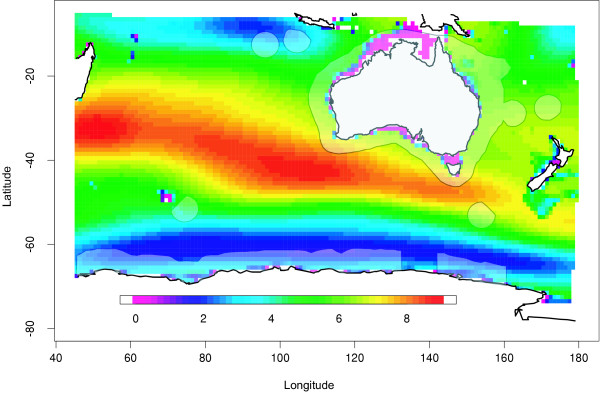
The estimated storage of anthropogenic CO_2 _(mol/m^2^) between 1990 and 1999 within the South Indian/Pacific Ocean. The approximate location of Australia's EEZ is shown in the shaded regions and includes Australia's continental EEZ, Norfolk and Lord Howe Islands in the Tasman Sea, Cocos and Christmas Islands in the sub-tropical Indian Ocean, the sub-Antarctic Islands (Macquarie and Heard) and the Australian Antarctic Territory. The total inventory of anthropogenic CO_2 _within the EEZ is 2.1 ± 0.7 PgCO_2_.

I have calculated the accumulation (storage) of anthropogenic CO_2 _within Australia's EEZ (including the Antarctic territory) for the 1990–1999 period to be 2.12 ± 0.7 Pg CO_2 _(Pg = 1 × 10^15 ^g) with an annual increase of about 220 Mt CO_2_/yr (Figure 1). The calculations are described in the methods section a the end of the manuscript. This storage of anthropogenic CO_2 _within Australia's EEZ does not necessarily imply the flux occurred within the EEZ as the ocean can redistribute CO_2 _from its uptake region to other locations. If I assume that Australia's EEZ absorbs anthropogenic CO_2 _at a rate equivalent to the global oceanic mean flux (7.3 ± 01.5 Pg CO_2_/yr [1,5]), I can obtain an independent flux estimate. As Australia's EEZ accounts for 2.4% of the total surface ocean (3.61 × 10^8 ^km^2^), the mean anthropogenic CO_2 _flux for Australia's ocean is about 175 MtCO_2_/yr throughout the 1990s. This calculation assumes Australia's EEZ acts in proportion to the global average oceanic anthropogenic CO_2 _flux. Even though the ocean is relatively homogenous, most studies suggest the Southern Ocean to be the region of highest uptake [6]. Since Australia's EEZ contains both Southern Ocean waters and sub-topical waters, it is likely that large variations occur within the Australian EEZ. Despite this however, my estimated range for anthropogenic CO_2 _uptake within the Australian EEZ (175–220 MtCO_2_/yr) is in agreement with a recent modelling study [7] that estimates a range between 160 to 340 MtCO_2_/yr depending on the areal extent of the Australian EEZ.

**Figure 2:**
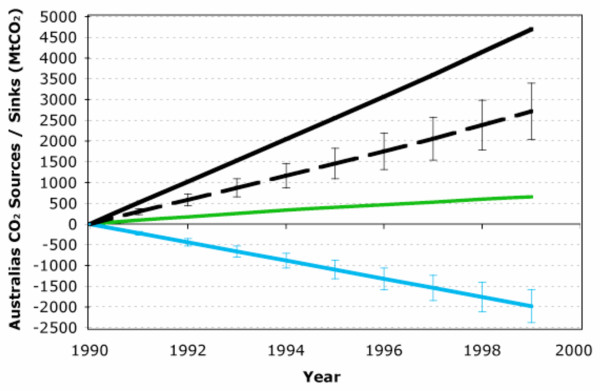
Anthropogenic CO_2 _budget for Australia during the 1990s. Fossil fuel CO_2 _emissions (shown by the solid black line) and land-use based emissions (shown by the solid green line) were taken from the Australian Greenhouse Office's National Carbon Accounting System. The estimated EEZ CO_2 _sink from this study is shown by the solid blue line with an associated uncertainty of approximately 20%. The sum of all these sources and sinks represent Australia's net CO_2 _emissions (shown by the dashed black line).

Australia's EEZ anthropogenic CO_2 _uptake is significant when comparing to Australia's CO_2 _emissions via fossil-fuel usage or land-use (Figure 2). Based on my analysis, Australia's oceanic EEZ CO_2 _sink over the 1990s (1750–1980 MtCO_2_) is about 3 times the magnitude of the CO_2 _source due to land-use changes (655 MtCO_2_) and about 30–40% of the total magnitude of fossil-fuel emissions (4695 MtCO_2_). The implications of including Australia's EEZ or any other nations EEZ within the framework of global carbon trading would be considerable. Furthermore direct human influence of the EEZ CO_2 _sink through carbon runoff from land use/irrigation/agricultural practices may also significantly influence national carbon accounts for nations with large EEZs.

Nations with large EEZs (like the USA) aren't necessarily those who will benefit the most from including the EEZ carbon sink in international climate policy, as it depends on the relative amount of EEZ sink in comparison to a nations annual anthropogenic emissions. Despite the USA claiming the worlds largest oceanic territory (~10 × 10^6 ^km^2^), its EEZ anthropogenic CO_2 _sink only absorbs less than 3% of its annual fossil-fuel emissions[8]. On the other hand small island nations (such as in the South Pacific) have the most to gain due to their low very anthropogenic CO_2 _emissions relative to their large potential EEZ CO_2 _sink. Tonga, Fiji, Samoa, Soloman Islands for example have vast oceanic territories that absorb many times over their annual fossil-fuel CO_2_emissions. Although Australia emits near the highest amount of anthropogenic CO_2 _per capita in the world, its relatively low population and vast oceanic territory results in the EEZ carbon sink being highly influential to its national carbon accounting. However, this 'natural anthropogenic CO_2 _sink' could be used as a disincentive for certain nations to reduce their anthropogenic CO_2 _emissions, which would ultimately dampen global efforts to reduce atmospheric CO_2 _concentrations. Along with the fact that the oceanic anthropogenic CO_2 _sink has little ability to be controlled by human activities, it should be explicitly excluded within current or future climate change policies. The international legality of the EEZ carbon sink and its potential implications requires careful consideration in formulating an equitable future framework for climate policy that aims at reducing atmospheric CO_2 _levels.

## Conclusion

The global EEZ represents over a quarter of the surface area of the ocean, which undoubtedly acts as important reservoir for sequestering anthropogenic CO_2_. Just as nation states have varying degrees of land coverage they also have varying degrees of EEZ extents. To demonstrate the potential implications of the EEZ anthropogenic CO_2 _sink, I have roughly estimated the uptake for Australias EEZ which is one of the largest in the world. By comparing the amount of anthropogenic CO_2 _sequestered by Australias EEZ to Australias CO_2 _emissions via fossil-fuel/land-use, I show that including the EEZ has significant implications for Australias national carbon accounts and any other nation who maintains a large EEZ. As the EEZ carbon sink may introduce legal grounds for nation states to possibly exploit, which would ultimately dampen efforts to reduce atmospheric CO_2 _concentrations, current and future international climate change policies should have an explicit 'EEZ' clause excluding its use within national carbon accounts.

## Methods

Due to the lack of temporal CO_2 _measurements within Australia's EEZ, to quantify the EEZ anthropogenic CO_2 _sink I use a recently developed method that exploits a purely transient tracer [1]. The method uses oceanic measurements of chlorofluorocarbons (CFC) coupled with atmospheric CFC observations [9] to determine water mass ages [10]. These water mass ages are then used with knowledge of the CO_2 _atmospheric history [11], alkalinity and carbonate chemistry equations [12] to estimate an accumulation of anthropogenic CO_2 _from 1990 to 1999. Although the method is indirect, the total uncertainty has been quantified to be between 10–20% by comparing results from direct temporal CO_2 _estimates [13] and within a general ocean circulation model [1].
